# Supplementary material for: Rapid Discrimination and Prediction of Ginsengs from Three Origins Based on UHPLC-Q-TOF-MS Combined with SVM
Source: Molecules. 2022 Jun 30;27(13):4225. doi: 10.3390/molecules27134225 (PMC9268438; doi:10.3390/molecules27134225)
Supplement: Supplementary file 1 [file molecules-27-04225-s001.zip › molecules-1757989-supplementary.pdf]

Supplementary Materials

Rapid Discrimination and Prediction of Ginsengs from Three Origins Based on UHPLC-Q-TOF-MS Combined with SVM

Chi Zhang [1,2,†], Zhe Liu [1,3,†], Shaoming Lu [1,2], Liujun Xiao [1,2], Qianqian Xue [1,2,*], Hongli Jin [1,2,*], Jiapan Gan [1,2,]
Xiaonong Li [2], Yanfang Liu [1,2] and Xinmiao Liang [1,2]

1 CAS Key Laboratory of Separation Science for Analytical Chemistry, Dalian Institute of Chemical Physics, Chinese Academy of Sciences, Dalian 116023, China; zhangchi@jcmsc.cn (C.Z.);liuzhe@dicp.ac.cn (Z.L.); lushaoming@jcmsc.cn (S.L.); xiaoliujun@jcmsc.cn (L.X.); ganjiapan@jcmsc.cn (J.G.); liuyanfang@jcmsc.cn (Y.L.); liangxm@dicp.ac.cn (X.L.)

2 Jiangxi Provincial Key Laboratory for Pharmacodynamic Material Basis of Traditional Chinese Medicine, Ganjiang Chinese Medicine Innovation Center, Nanchang 330000, China;
lixiaonong@jcmsc.cn

3 University of Chinese Academy of Sciences, Beijing 100049, China

* Correspondence: xueqianqian@jcmsc.cn (Q.X.); jinhongli@jcmsc.cn (H.J.)

† These authors contributed equally to this work.

Figure S1 Optimization of UHPLC-MS analysis conditions. (A: the optimization of extraction solvents , B: the optimization of mobile phase (mobile phase 1: water-acetonitrile, mobile phase 2: water (contain 0.01% formic acid)-acetonitrile (contain 0.01% formic acid) , C: the optimization of elution gradient (Gradient 1: 0~10 min, 19% B; 10~16 min, 19%~28% B; 16~35 min, 28~35% B, Gradient 2: 0~8 min, 19% B; 8~13 min, 19%~21% B; 13~14 min, 21%~25%; 14~20 min, 25%~29% B; 20~34 min, 29% B; 34~35 min, 29%~32% B; 35~42 min, 32%~40% B, Gradient 3: 0~10 min, 19% B; 10~16 min, 19%~28% B; 16~30 min, 28~34% B; 30~31 min, 34%~90% B; 31~35 min, 90%~90% B. D: the optimization of injection concentration).

Figure S2 TIC of ginseng sample by UHPLC-Q-TOF-MS(Take a QC sample as an example).

Figure S3 The MS/MS spectra of ginsenosides: (A)PPT, protopananxatriol-type; (B)PPD, protopanaxadiol-type; (C)OA, oleanane-type.

Figure S4 The full scan mode and MS/MS mode of six compounds, including Compound 13(ginsenoside Rg1), Compound 14(ginsenoside Re), Compound 27(ginsenoside Rf), Compound 46(ginsenoside Rb1), Compound 50(ginsenoside Rc), Compound 56(ginsenoside Rb2).

Figure S5 A presentation of 200 times permutation test for PLS-DA analysis.

Figure S6 Distribution of six peaks in ginseng from three different origins.

Table S1 Stability and repeatability of UHPLC-Q-TOF-MS.

Table S2 The classified and predicted results of ginsengs from three geographical origins using SVM model six quality markers.

In order to achieve good separation effect and obtain high-quality UHPLC-Q-TOF-MS data, we optimized the extraction solvents, composition of mobile phase, elution gradient and injection concentration in detail during UHPLC-Q-TOF-MS analysis.

Firstly, different extraction solvents, including methanol, 70% methanol, 40% ethanol and ethanol, were investigated to achieve maximum extraction. The results showed that the 40% ethanol is more suitable for the extraction of ginseng because of its high extraction efficiency and environmental protection (Figure S1, A). Additionally, the chromatographic conditions including mobile phase and elution gradient were optimized to increase the separation. Consequently, water (contain 0.01% formic acid, v:v) -acetonitrile (contain 0.01% formic acid, *v*:*v*) and gradient 3 (0 ~ 10 min, 19% B; 10 ~ 16 min, 19% ~ 28% B; 16 ~ 30 min, 28 ~ 34% B; 30 ~ 31 min, 34% ~ 90% B; 31 ~ 35 min, 90% ~ 90% B.) were preferred as the most suitable mobile phase system and elution gradient due to more peak numbers and better resolution (Figure S1, B~C). Finally, the chromatographic conditions was used to gather the UHPLC-Q-TOF-MS data, and the injection concentration of 20000 ppm can obtain excellent response and will not burden the instrument by comparing the spectral profile 20 ppm, 10000 ppm and 20000 ppm (Figure S1, D).

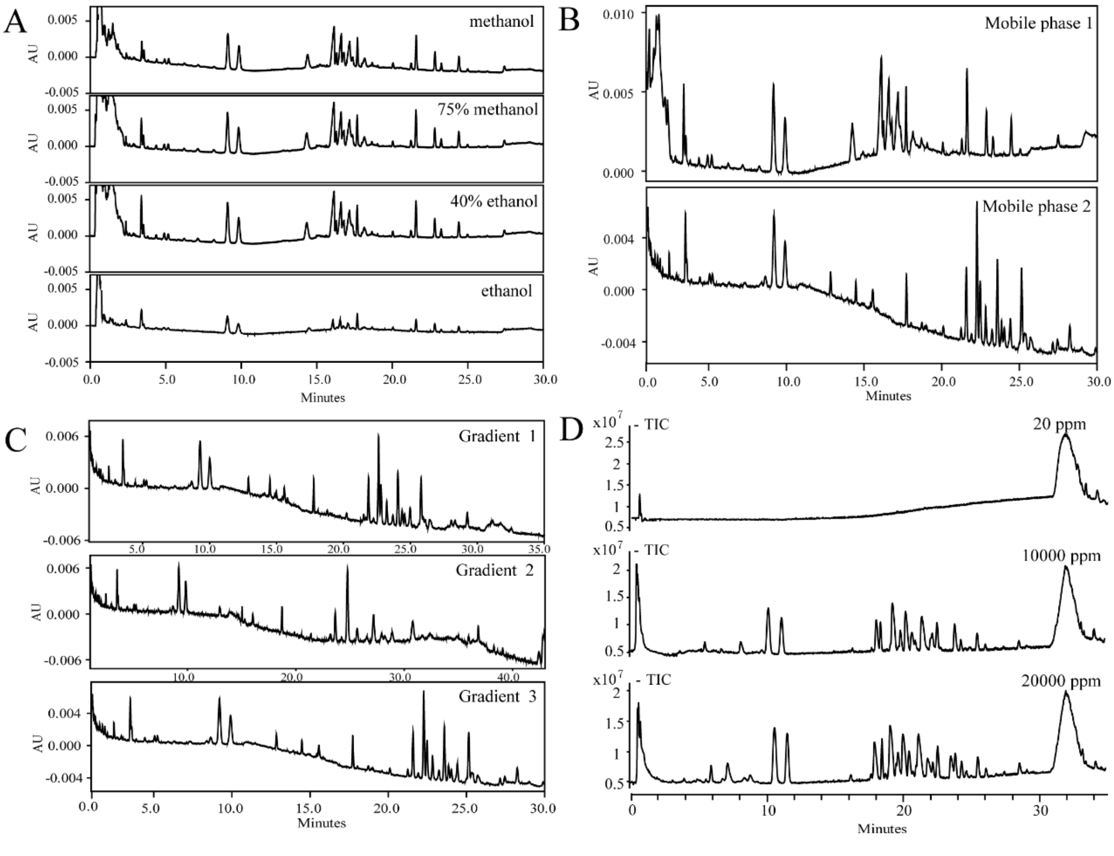

Figure S1. Optimization of UHPLC-MS analysis conditions. (**A**: the optimization of extraction solvents , **B**: the optimization of mobile phase (mobile phase 1: water- acetonitrile, mobile phase 2: water (contain 0.01% formic acid)-acetonitrile (contain 0.01% formic acid) , **C**: the optimization of elution gradient (Gradient 1: 0~10 min, 19% B; 10~16 min, 19%~28% B; 16~35 min, 28~35% B, Gradient 2: 0~8 min, 19% B; 8~13 min, 19%~21% B; 13~14 min, 21%~25%; 14~20 min, 25%~29% B; 20~34 min, 29% B; 34~35 min, 29%~32% B; 35~42 min, 32%~40% B, Gradient 3: 0~10 min, 19% B; 10~16 min, 19%~28% B; 16~30 min, 28~34% B; 30~31 min,

34%~90% B; 31~35 min, 90%~90% B. **D**: the optimization of injection concentration).

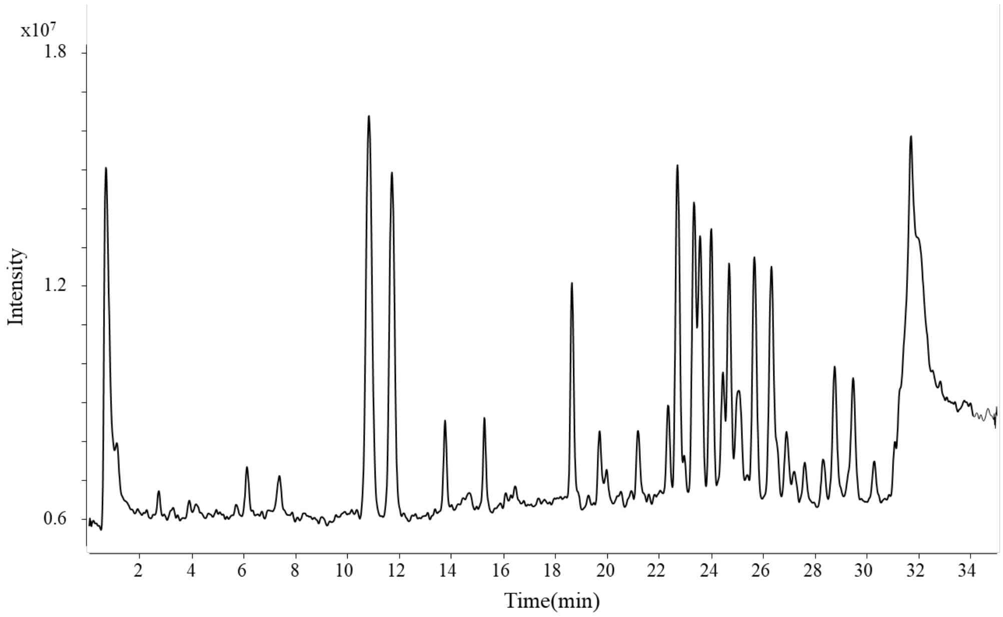

Figure S2. TIC of ginseng sample by UHPLC-Q-TOF-MS(Take a QC sample as an example).

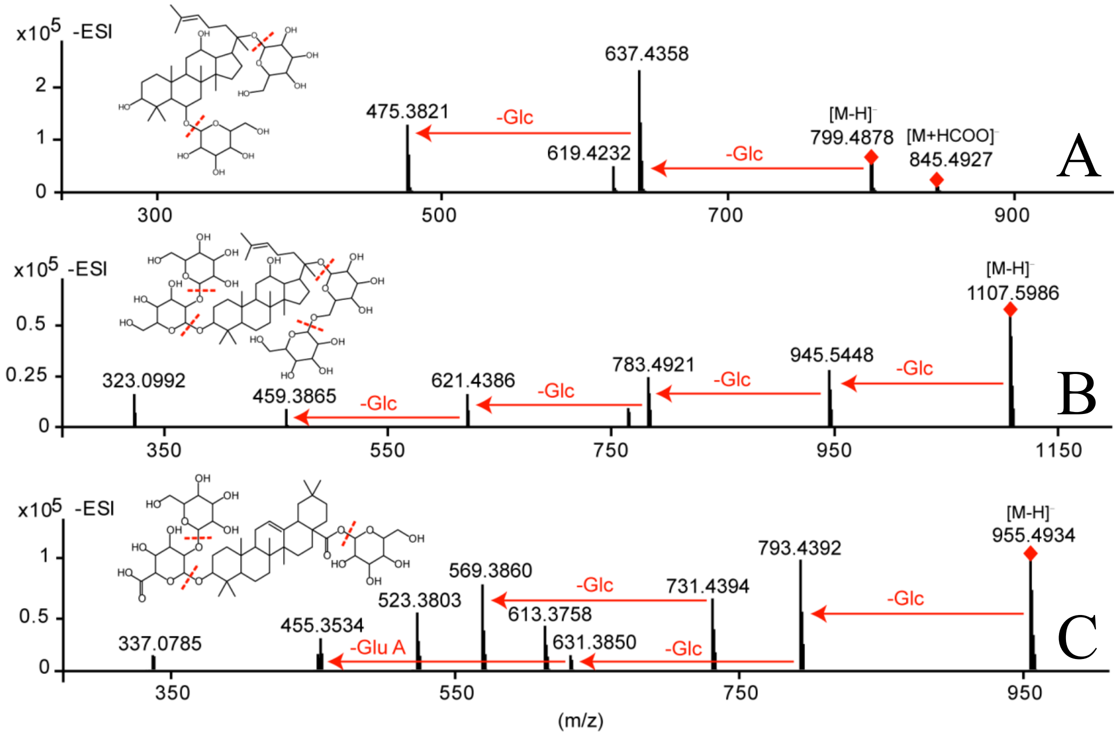

Figure S3. The MS/MS spectra of ginsenosides: (A)PPT, protopananxatriol-type; (B)PPD, protopanaxadiol-type; (C)OA, oleanane-type.

Compound 13(ginsenoside Rg1)

MS 845.4910[M+COOH]$^-$

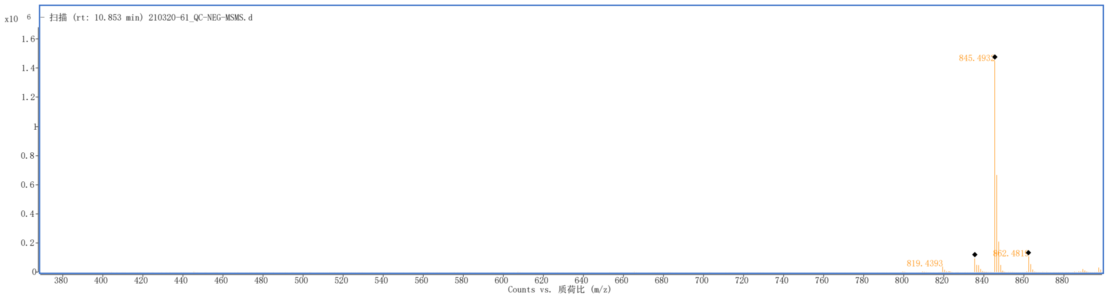

MS/MS 799.4852[M-H]$^-$; 637.4337[M-H-Glc]$^-$; 619.4215[M-H-Glc-O]$^-$; 475.3802[M-H-Glc-Glc]$^-$

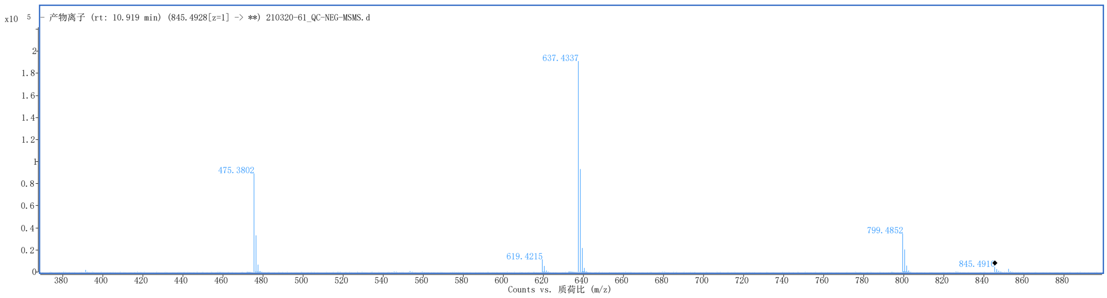

Compound 14(ginsenoside Re)

MS 945.5475[M-H]$^-$

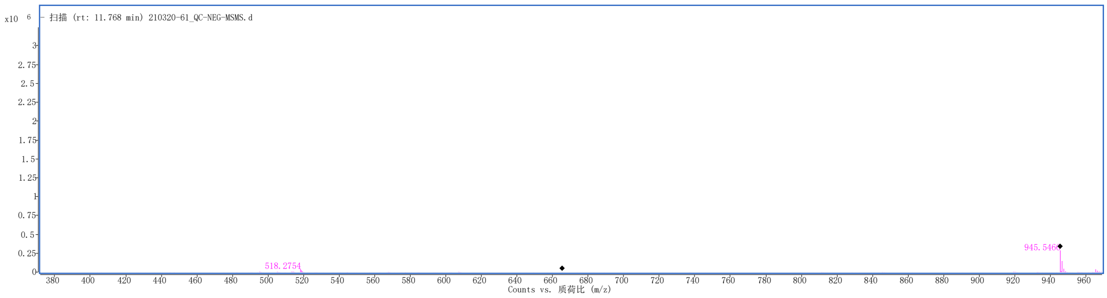

MS/MS 799.4880[M-H-Rha]$^-$; 783.4926[M-H-Glc]$^-$; 765.4816[M-H-Glc-H_2O]$^-$; 637.4346[M-H-Glc-Rha]$^-$; 619.4264[M-H-Glc-Rha-O]$^-$; 475.3818[M-H-Glc-Glc-Rha]$^-$

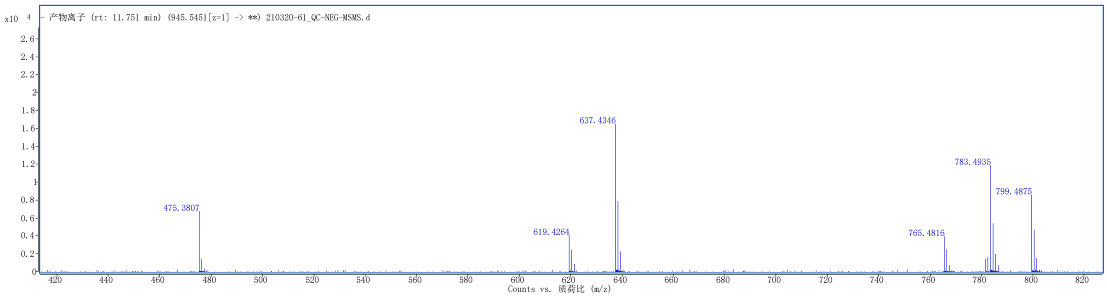

Compound 27(ginsenoside Rf)

MS 799.4873[M-H]$^-$

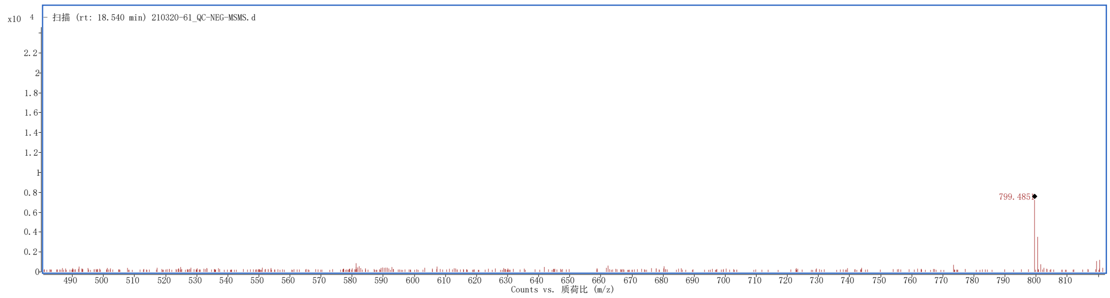

MS/MS 637.4327[M-H-Glc]$^-$; 475.3796[M-H-Glc-Glc]$^-$

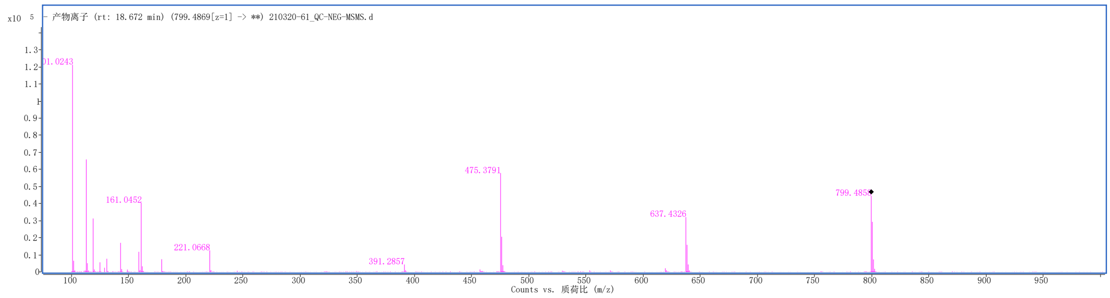

Compound 46(ginsenoside Rb1)

MS 1107.6022[M-H]$^-$;

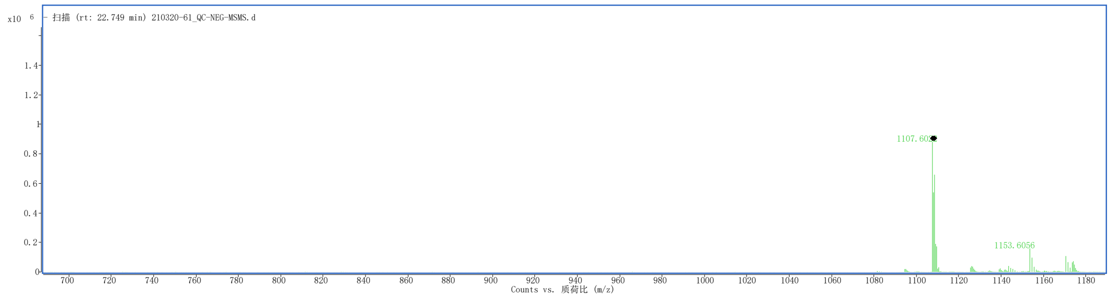

MS/MS 945.5438[M-H-Glc]$^-$; 783.4904[M-H-Glc-Glc]$^-$

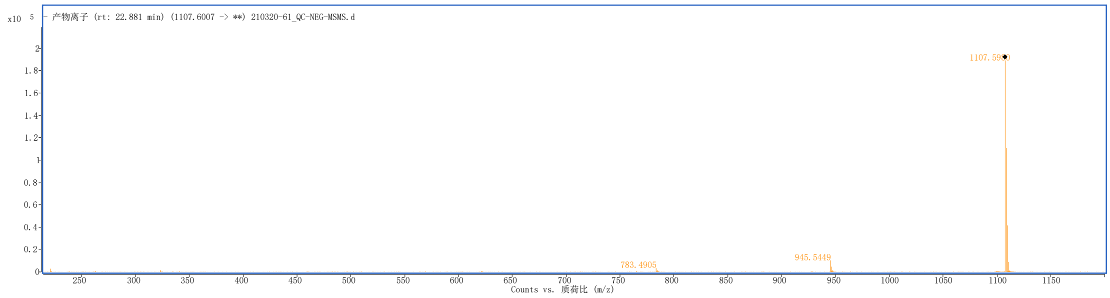

Compound 50(ginsenoside Rc)

MS 1077.5897[M-H]-

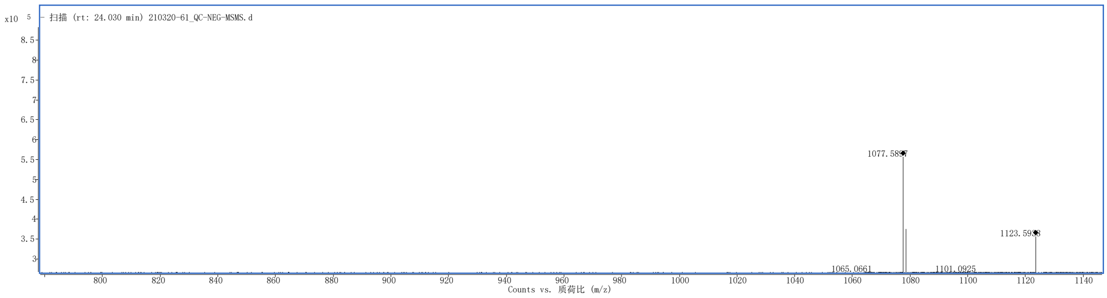

MS/MS 945.5412[M-H-Ara]-; 783.4910[M-H-Ara-Glc]-

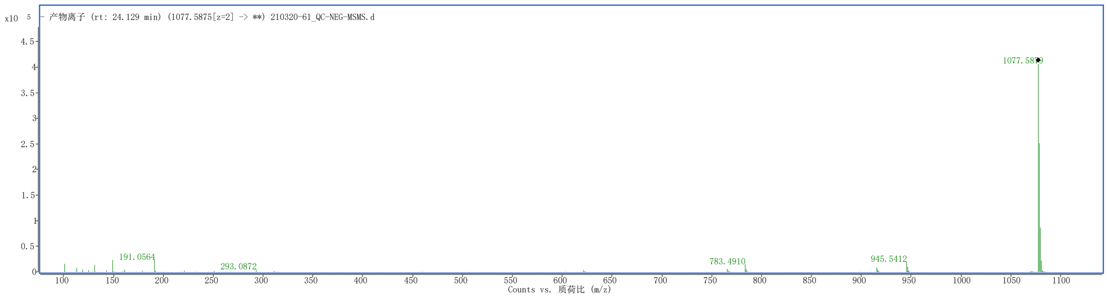

Compound 56(ginsenoside Rb2)

MS 1077.5859 [M-H]-

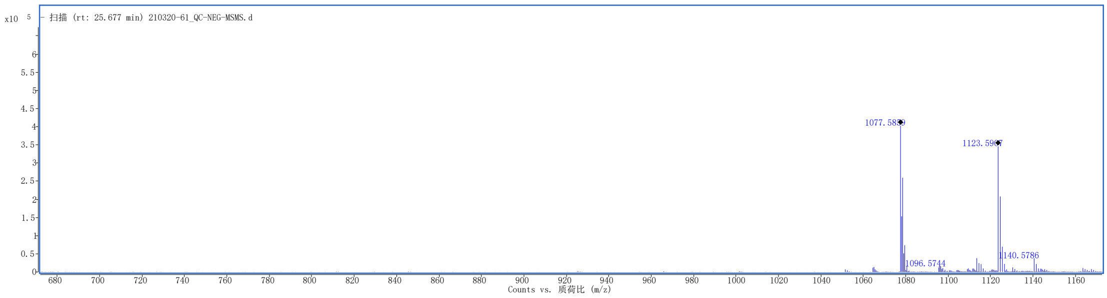

MS/MS 945.5412[M-H-Ara]-; 783.4910[M-H-Ara-Glc]-

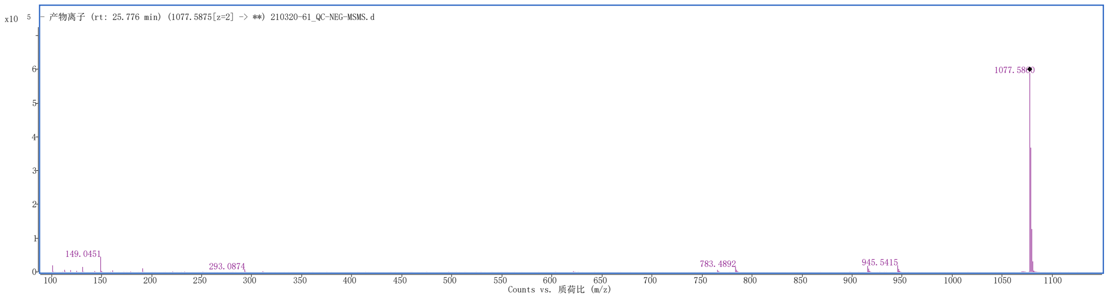

Figure S4. The full scan mode and MS/MS mode of six compounds, including Compound 13(ginsenoside Rg1), Compound 14(ginsenoside Re), Compound 27(ginsenoside Rf), Compound 46(ginsenoside Rb1), Compound 50(ginsenoside Rc), Compound 56(ginsenoside Rb2).

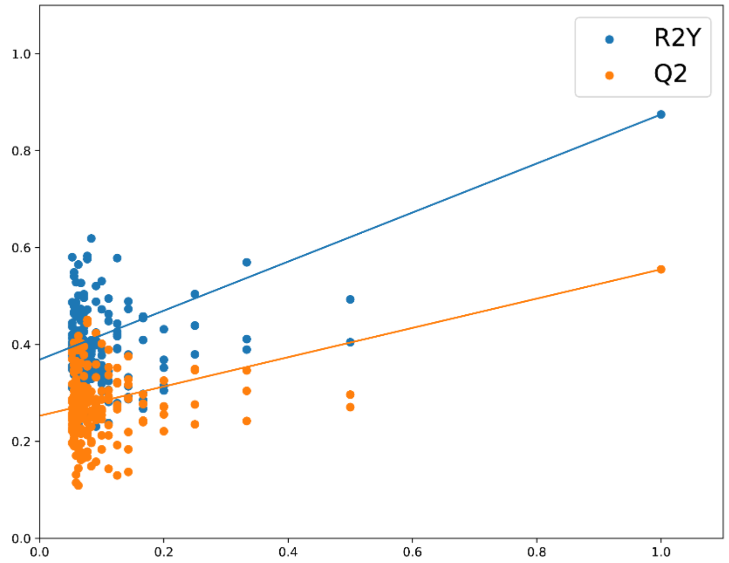

Figure S5. A presentation of 200 times permutation test for PLS-DA analysis.

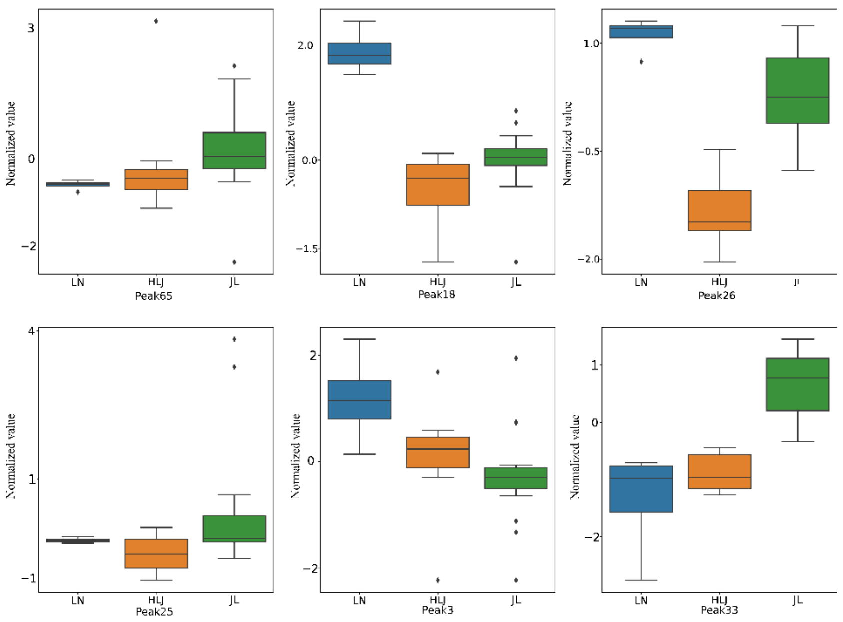

Figure S6. Distribution of six peaks in ginseng from three different origins.

Table S1. Stability and repeatability of UHPLC-Q-TOF-MS.

		QC-2	QC-13	QC-19	QC-25	QC-31	QC-37	QC-43	QC-49	QC-55	QC-60	RSD (%)
peak 13(Rg1)	MS acurracy	800.49298	800.49308	800.49546	800.49704	800.49495	800.49508	800.49538	800.49508	800.49471	800.49517	1.46×10^{-4}
	RT(min)	10.83	10.81	10.85	10.87	10.81	10.86	10.93	10.95	10.85	10.89	4.32×10^{-1}
	Peak area	104954316	98481490	104232277	103086926	102544917	101768074	100029418	99264788	99795025	99269551	2.25
peak 14(Re)	MS acurracy	946.55122	946.55109	946.55367	946.55462	946.55343	946.55311	946.55436	946.55390	946.55415	946.55340	1.29×10^{-4}
	RT(min)	11.70	11.70	11.72	11.74	11.71	11.74	11.78	11.79	11.71	11.75	2.73×10^{-1}
	Peak area	76607661	72648717	77489582	76135696	76523550	75786941	74393491	73026515	74342989	73553248	2.23
peak 27 (Rf)	MS acurracy	800.49304	800.49290	800.49574	800.49440	800.49524	800.49504	800.49409	800.49520	800.49403	800.49498	1.19×10^{-4}
	RT(min)	18.65	18.65	18.64	18.64	18.63	18.66	18.67	18.66	18.65	18.66	6.42×10^{-2}
	Peak area	28790682	27441278	29390796	29296699	28856501	28822708	28470863	28194414	28299646	28334538	2.00
peak 46(Rb1)	MS acurracy	1108.60430	1108.60445	1108.60796	1108.60701	1108.60586	1108.60786	1108.60783	1108.60636	1108.60730	1108.60627	1.21×10^{-4}
	RT(min)	22.70	22.70	22.70	22.70	22.71	22.75	22.74	22.73	22.73	22.75	9.38×10^{-2}
	Peak area	19099289	18536386	19789290	19433051	19622146	19474279	19308590	18868514	19080411	19071874	1.94
peak 50(Rc)	MS acurracy	1078.59314	1078.59310	1078.59548	1078.59522	1078.59588	1078.59617	1078.59575	1078.59556	1078.59689	1078.59591	1.15×10^{-4}
	RT(min)	24.01	24.00	24.02	24.01	24.00	24.05	24.07	24.06	24.05	24.06	1.14×10^{-1}
	Peak area	17472904	17019343	18109570	18052868	18026347	18050858	17712371	17466715	17514756	17685107	2.00
peak 56(Rb2)	MS acurracy	1078.59327	1078.59312	1078.59509	1078.59566	1078.59684	1078.59574	1078.59735	1078.59487	1078.59543	1078.59654	1.29×10^{-4}
	RT(min)	25.67	25.66	25.68	25.66	25.66	25.71	25.72	25.71	25.70	25.73	1.05×10^{-1}
	Peak area	19476807	18557294	19919615	20125461	20073028	19841236	19583815	19527324	19468198	19524613	2.27
peak 64(Rd)	MS acurracy	946.55111	946.55088	946.55230	946.55242	946.55295	946.55269	946.55357	946.55296	946.55355	946.55412	1.10×10^{-4}
	RT(min)	28.76	28.72	28.78	28.74	28.75	28.79	28.79	28.79	28.79	28.82	1.04×10^{-1}
	Peak area	22393423	21322836	23145705	23057876	23148229	23055027	22538266	22461615	22836546	22785177	2.44

Table S2. The classified and predicted results of ginsengs from three geographical origins using SVM model six quality markers.

six quality markers (accuracy=100%)					
Sample	Actual	Recognized	Sample	Actual	Recognized
S1	LN	LN	S17	JL	JL
S2	LN	LN	S18	JL	JL
S3	LN	LN	S19	JL	JL
S4	LN	LN	S20	JL	JL
S5	HLJ	HLJ	S21	JL	JL
S6	HLJ	HLJ	S22	JL	JL
S7	HLJ	HLJ	S23	JL	JL
S8	HLJ	HLJ	S24	JL	JL
S9	JL	JL	S25	JL	JL
S10	JL	JL	S26	JL	JL
S11	JL	JL	S27	JL	JL
S12	JL	JL	S28	JL	JL
S13	HLJ	HLJ	S29	JL	JL
S14	HLJ	HLJ	S30	JL	JL
S15	HLJ	HLJ	S31	JL	JL
S16	HLJ	HLJ			
